# Recent Advances in the Synthesis of the Marine-Derived Alkaloid Fascaplysin and Its Metabolites Homofascaplysins A–C

**DOI:** 10.3390/molecules29071590

**Published:** 2024-04-02

**Authors:** Ramana Reddy Mittapalli, Harshita Kumari

**Affiliations:** James L. Winkle College of Pharmacy, University of Cincinnati, Cincinnati, OH 45267-0514, USA

**Keywords:** marine natural products, sponge, fascaplysin, homofascaplysins A, B, and C, alkaloids

## Abstract

The fascaplysin and homofascaplysin class of marine natural products has a characteristic 12H-pyrido[1,2-a:3,4-b′]diindole pentacyclic structure. Fascaplysin was isolated in 1988 from the marine sponge *Fascaplysinopsis bergquist* sp. The analogs of fascaplysin, such as homofascaplysins A, B, and C, were discovered late in the Fijian sponge F. reticulate, and also have potent antimicrobial activity and strong cytotoxicity against L-1210 mouse leukemia. In this review, the total synthesis of fascaplysin and its analogs, such as homofascaplysins A, B, and C, will be reviewed, which will offer useful information for medicinal chemistry researchers who are interested in the exploration of marine alkaloids.

## 1. Introduction

**Figure 1:**
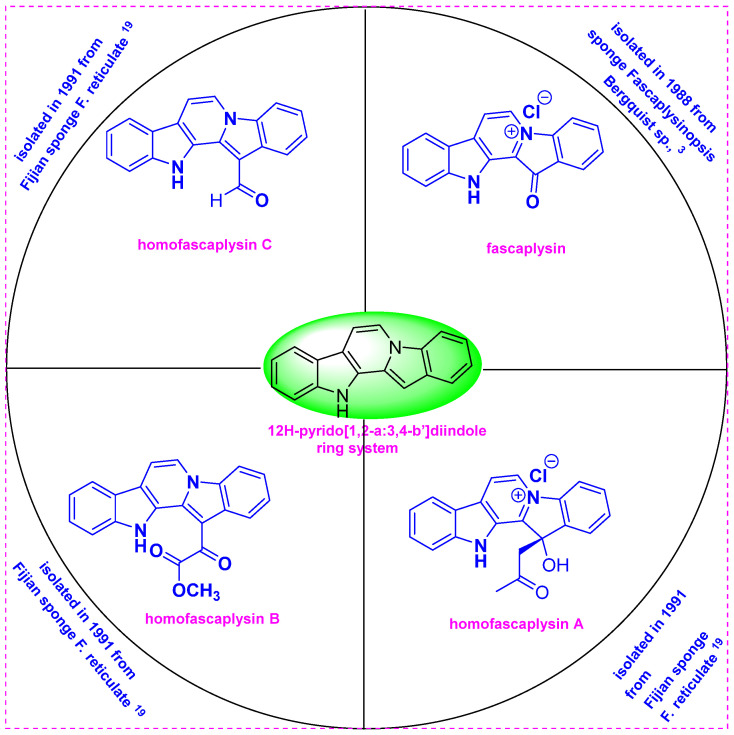
Structures of fascaplysin and homofascaplysins A–C.

The β-carboline group is a valuable structural motif found in many plants, marine organisms, insects, and mammals, and compounds in this family show a broad range of biological activities [1,2,3,4]. In particular, the 12H-pyrido[1,2-a:3,4-b′]diindole ring system is an important component of various marine alkaloids, such as fascaplysin and homofascaplysins A–C (Figure 1) [5]. Among compounds derived from the sponge *Fascaplysinopsis bergquist* sp., fascaplysin, which was first isolated in 1988, is the most studied [6]. This natural product was later isolated by several other researchers from *Hyrtios erecta* [7] as well as from *Thorectandra* sp. *tunicate* [8], *Didemnum* sp. [9]., and sponge *Fascaplysinopsis* [10].

In 1988, Ireland et al. first isolated and determined the structure of the red pigment fascaplysin, a pentacyclic quaternary salt from the Fijian sponge *Fascaplysinopsis bergquist* sp., which was collected near Dravuni Island, Fiji [6]. The lyophilized sponge was extracted with methanol, and then fractionation was performed using the Kupchan partitioning method. The above methanol/water fraction was washed with CCl_4_ and then extracted with chloroform. This CHCl_3_ partition exhibited substantial cytotoxicity and high antimicrobial activity. The chloroform fraction was chromatographed over Sephadex LH-20 using methanol, and subsequent purification by crystallization from methanol yielded fascaplysin. The structure of fascaplysin (13-oxo-12,13-dihydropyrido[1,2-a:3,4-b′] diindol-5-ium chloride) was determined using ^1^H and ^13^C NMR spectroscopy, X-ray analysis, and El and FAB mass spectrometry (Figure 1).

In 1990, Crews et al. isolated the bioactive constituents from *F. reticulata* collected from the Benga Lagoon, Fiji. *F. reticulata* was found to contain new 12H-pyrido[1,2-a:3,4-b′]diindole alkaloids such as homofascaplysins A–C as well as the known natural products fascaplysin and (+)-octopamine [11]. Four separate specimens of F. reticulata sponge were collected by SCUBA at 10–20 m between 1985 and 1989 from the Benga Lagoon, Fiji. The specimens were preserved for long periods before extraction. The viscous crude oil obtained by methanol extraction of the sponge specimens contained three alkaloids as major constituents, as determined by ^1^H and ^13^C NMR spectroscopy. The crude oil was then successively partitioned using equal volumes of aqueous methanol and a series of solvents including hexanes, CCl_4_, and dichloromethane. The CCl_4_ fraction was chromatographed over Sephadex LH_2_0 using methanol and then purified by repeated reversed-phase HPLC. This fraction afforded a mixture of homofascaplysins A–C.

Fascaplysin inhibits the growth of several microbes [12] and has been widely studied for the selective inhibition of CDK4, a cyclin-dependent kinase (CDK), as reported by Sony et al. [13]. The inhibition of CDKs by small molecules is an area of recent interest in anticancer research [14]. Fascaplysin is the first biologically active planar, toxic anticancer agent. Unfortunately, fascaplysin cannot be used as an anticancer drug because it is extremely toxic due to its planar structure that intercalates with DNA [15]. As a key element in the cell division cycle, CDKs are essential for cell proliferation and healthy cell growth. Consequently, various nonplanar analogs of fascaplysin have been synthesized and studied. Amongst these compounds, the CDK4 inhibitors BPT and CA224 inhibit tubulin polymerization in vitro and exhibit antitumor activity in the colon cancer tumor model HCT-116 in vivo [16].

Chen et al. revealed that fascaplysin inhibits angiogenesis by suppressing the vascular endothelial growth factor (VEGF) and inducing apoptosis in human umbilical vein endothelial cells (HUVECs) [17]. In addition, Oh et al. demonstrated that fascaplysin inhibits VEGFR2, TRKA, HIF-1α, and survivin, thereby preventing cancer cell growth [18]. Hamilton et al. examined the cytotoxic effects of fascaplysin against non-small cell and small cell lung cancer. Fascaplysin was found to induce tumor cell apoptosis through several mechanisms, including G1/0 cell cycle arrest [19]. Furthermore, fascaplysin significantly upregulates the expression of PD-L1 in lung cancer cells, thus improving the sensitivity of anti-PD-1 immunotherapy in vivo.

The enzyme acetylcholinesterase (AChE) is responsible for the death of neurons in Alzheimer’s disease. Fascaplysin inhibits AChE noncompetitively, with IC50 and Ki values of 1.49 and 2.28 µM, respectively [20]. At 1 mM, fascaplysin displays promising P-gp activation and AChE inhibition, which together with good medication safety demonstrates the potential of this compound as an anti-Alzheimer agent [21]. Furthermore, fascaplysin shows potential antiplasmodial activity, inhibiting *Plasmodium falciparum* strains NF54 and K1 with IC50 values of 34 and 50 ng/mL, respectively [22]. Thus, fascaplysin is a potent in vitro inhibitor of chloroquine-resistant *P. falciparum* and chloroquine-susceptible (NF54) strains. Due to the potent antiplasmodial activity, fascaplysin demonstrates the potential to be a leading structure. In addition, the fascaplysin analogs homofascaplysins A–C, which were isolated from the Fijian sponge Fascaplysinopsis reticulate in 1991, have strong cytotoxicity against L-1210 mouse leukemia cells and potent antimicrobial activity [11].

Although fascaplysin has a wide-ranging bioactive spectrum, its advance study has been disturbed by the limited number of compounds isolated from marine microorganism sources. In the future, modifying the planar structure of fascaplysin to reduce its toxicity could play a huge role in chemistry discoveries. These encouraging biological activities linked with fascaplysin have already led to the discovery of a few synthetic targets, and there is much unexplored medicinal chemistry space that may further lead to the discovery of novel biological active lead compounds. Investigations on nonplanar fascaplysin derivatives can form the starting point for elaboration of novel lead compounds, and therefore require finding effective procedures for their syntheses. As a result, many research groups have enthusiastically put their effort into total synthesis. This review may help to find the suitable total synthesis method for a nontoxic, nonplanar structure of fascaplysin derivatives in future synthesis.

Many methods have been established for the synthesis of fascaplysin, but there are limited reports on the synthesis of homofascaplysins B and C. Moreover, to the best of our knowledge, the synthetic methods for obtaining fascaplysin and related analogs, such as homofascaplysins A–C, have not yet been comprehensively reviewed. Herein, we systematically review important advances in synthetic strategies for obtaining fascaplysin and its metabolites homofascaplysins A–C, focusing on a selection of representative examples.

## 2. Discussion

### 2.1. Total and Formal Synthesis of Fascaplysin

**Scheme 1:**
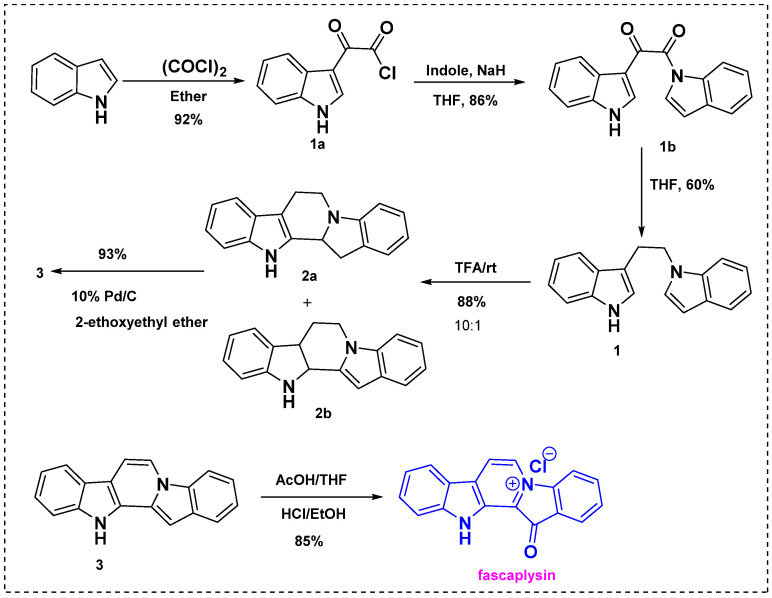
First total synthesis of fascaplysin by Gribble et al. [23].

In 1990, Gribble et al. described the first total synthesis of fascaplysin from indole (Scheme 1) [23]. This approach involved peracid oxidation followed by reaction with oxalyl chloride/methanol or Vilsmeier formylation of the key diindole intermediate. Initially, the indole was treated with oxalyl chloride in ether and gave 3-indolyl glyoxylyl chloride **1a** at 92%, as shown in Scheme 1. Then, 3-indolylglyoxylyl chloride **1a** was permitted to react with I-indolylsodium to give keto amide **1b** in an 86% yield. The next step was reduction of **1b** with sodium (mono)trifluoroacetoxyborohydride, which afforded diindole **1** in a 60% yield and a 47% overall yield from the starting indole. Further, diindole **1** was then treated with trifluoro acetic acid to obtain a mixture of cyclized products **2a** and **2b** in a 10:1 ratio. Direct oxidation of this mixture with 10% Pd/C in refluxing 2-ethoxyethyl ether gave fully aromatic pentacycle **3** as a pale green solid. The reaction of **3** with peracetic acid in cold THF followed by treatment with con. HCl/EtOH afforded fascaplysin in an 85% yield. Thus, fascaplysin was efficiently synthesized from indole in seven steps with an overall yield of 65%. The main advantage of this method is that none of the steps, except the final one, involve chromatography, and the overall yield was very high.

**Scheme 2:**
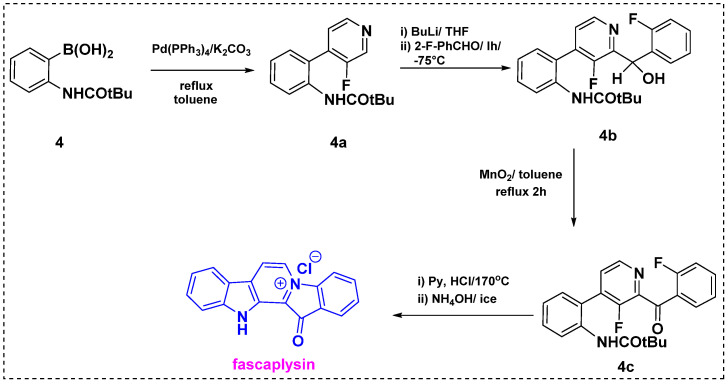
Total synthesis of fascaplysin by Qukguiner et al. [24].

In 1993, Qukguiner et al. developed a strategy for the total synthesis of fascaplysin from simple benzene and pyridine derivatives (Scheme 2) [24]. Palladium-catalyzed Suzuki cross-coupling between boronic acid **4** and iodopyridine gave bis aryl product **4a**. Regioselective coupling of **4a** with 2-fluorobenzaldehyde in the presence of n-BuLi afforded corresponding pyridine **4b** in a 95% yield. Further, oxidation of **4b** with MnO_2_ in refluxing toluene provided carbonyl derivative **4c** in a 99% yield. The one-pot double cyclization of **4c** by treatment with pyridinium chloride at 170 °C followed by a basic workup gave fascaplysin in an 82% yield. The reported synthesis of fascaplysin relies on key steps such as cross-coupling, metalation, and cyclization. This method is fully regioselective and provides a 76% overall yield from starting material **4**.

**Scheme 3:**
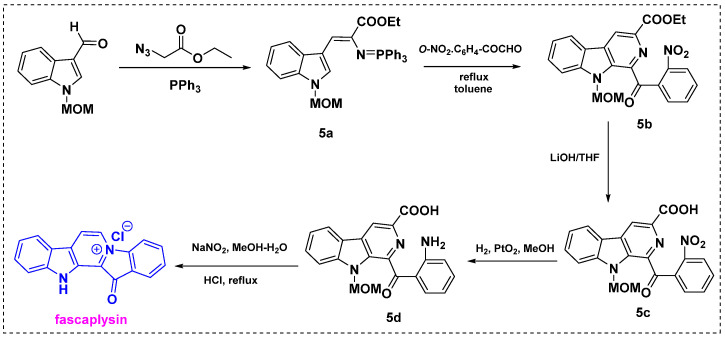
Total synthesis of fascaplysin by Molina et al. [25].

In 1994, Molina et al. described the synthesis of fascaplysin from *N*-methoxymethyl-3-formylindole in four steps (Scheme 3) [25]. *N*-Methoxymethyl-3-formylindole was converted into intermediate iminophosphorane **5a** by treatment with ethyl azidoacetate followed by triphenylphosphine. Iminophosphorane **5a** was treated with a nitro-substituted aryl glyoxal in toluene at 160 °C in a sealed tube to give 1-aroyl-β-carboline derivative **5b** in a 60–65% yield. Subsequent hydrolysis of **5b** with LiOH/THF at room temperature provided **5c** in a 90% yield. The nitro group was then selectively reduced by PtO_2_/catalytic hydrogenation to give **5d** in an 80% yield. Finally, deprotection and decarboxylation by diazotization and further heating afforded fascaplysin in a 60% yield. This method provides a 72% overall yield from the starting material.

**Scheme 4:**
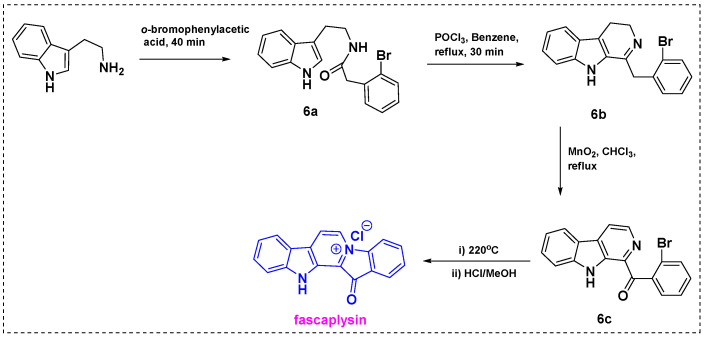
Total synthesis of fascaplysin from tryptamine.

In 1997, Novikov et al. obtained a 44% overall yield for the total synthesis of fascaplysin from tryptamine in five steps (Scheme 4) [26]. Acylation of tryptamine with o-bromophenylacetic acid gave corresponding amide **6a**, which was converted into dihydro-13-carboline **6b** using POCl_3_. Subsequent reaction of **6b** with MnO_2_ in CHCl_3_ under reflux conditions gave α-acyl-substituted β-carboline **6c**. Heating of **6c** yielded pyridodiindole quaternary salt **6d**, which was treated with dry HCl in methanol to give fascaplysin in an overall yield of 44%.

**Scheme 5:**
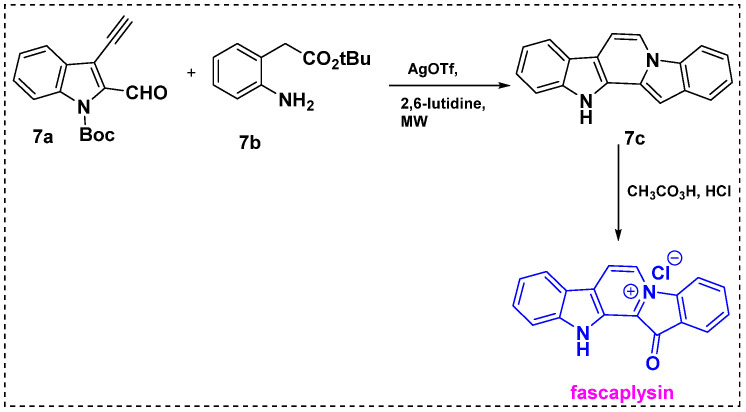
Total synthesis of fascaplysin via a silver-catalyzed cascade reaction.

In 2010, Waldmann et al. developed a silver-catalyzed cascade reaction, which was then applied in the total synthesis of fascaplysin (Scheme 5) [27]. In this method, commercially available Boc-protected 3-ethynyl-indole-2-carbaldehyde **7a** was used as a precursor. The microwave-assisted silver-catalyzed cascade cyclization of **7a** with aniline **7b** yielded pentacyclic core **7c** in a high yield after acidic workup. Peracetic acid oxidation of diindole **7c** followed by salt formation with con. HCl provided a 52% overall yield.

**Scheme 6:**
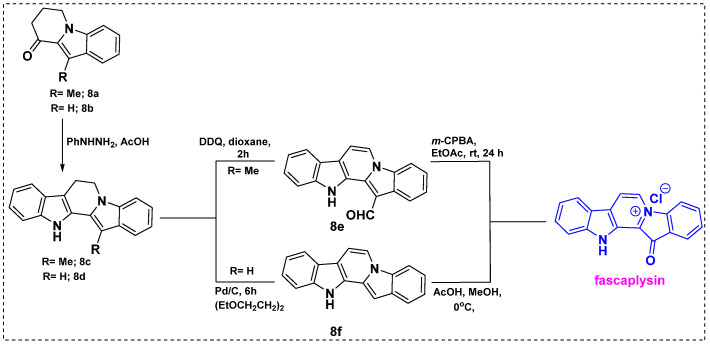
Total synthesis of fascaplysin using Fischer cyclization as a key step.

Also in 2010, Zhidkov et al. developed a short synthetic route to fascaplysin from 3-methyl indole/indole ketone 8a/8b using the popular Fischer indole synthesis reaction as a key step (Scheme 6) [28]. Fischer cyclization of **8a** or **8b** gave corresponding bisindole **8c** or **8d** in a 90–91% yield, which were further converted into **8e** by 2,3-dichloro-5,6-dicyanobenzoquinone (DDQ) oxidation or **8f** by dehydrogenation over Pd/C. The yield was 50% for **8e** and 75% for **8f**. Finally, the reaction of **8e** with mCPBA provided fascaplysin in a 67% yield and **8f** with acetic acid in methanol gave fascaplysin in 85% yields. Therefore, fascaplysin can be synthesized from commercially available ethyl indole-2-carboxylate in seven steps with overall yields of 69% through **8e** and 82% through intermediate **8f**.

**Scheme 7:**
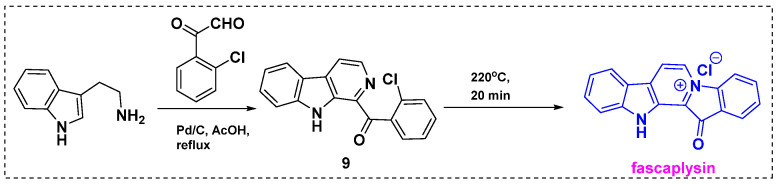
Total synthesis of fascaplysin from tryptamine.

In 2012, Bharate et al. described a two-step total synthesis of fascaplysin from commercially available tryptamine in a 68% overall yield (Scheme 7) [29]. As well as being shorter, less expensive, and more efficient than previously reported synthetic methods, this approach has potential for the large-scale synthesis of fascaplysin. A key step in this strategy is tandem dehydrative condensation between ortho-chloro-substituted glyoxal and tryptamine followed by dehydrogenation. Initially, tryptamine was reacted with glyoxal in acetic acid in the presence of Pd/C to give cyclized product **9** in an 85% yield. Next, β-carboline **9** was heated at 220 °C for 20 min to induce ring closure and produce fascaplysin in an 80% yield.

**Scheme 8:**
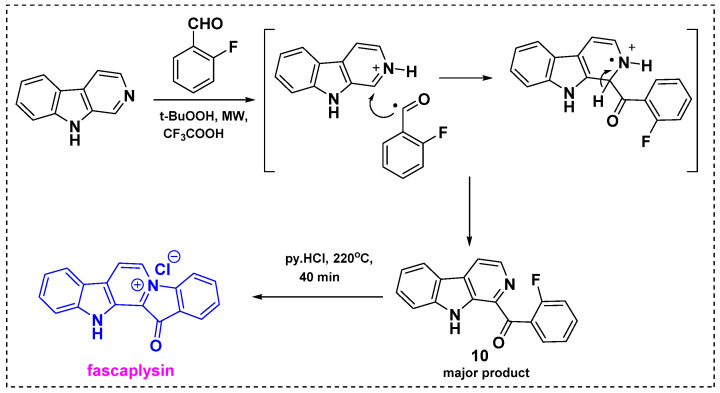
Two-step synthesis of fascaplysin under microwave conditions.

In 2013, Zhidkov et al. developed a two-step method for the synthesis of fascaplysin via a microwave-assisted Minisci reaction of β-carboline followed by intramolecular quaternization (Scheme 8) [30]. Initially, the reaction of β-carboline with o-fluorobenzaldehyde and t-BuOOH in TFA under microwave irradiation gave β-carboline **10** in a 65% yield as well as trace amounts of byproducts. This report presents the first microwave-assisted Minisci reaction. In this reaction, TFA can act as an acid catalyst and t-BuOOH is involved in the radical generation in the aldehyde moiety. Therefore, TFA was catalyzed to initiate nucleophilic radical substitution to an electron-deficient aromatic compound of β-carboline, resulting in the introduction of an alkyl group of the aldehyde moiety to a nitrogen containing β-carboline, as shown in Scheme 8. Heating of **10** with pyridinium chloride at 220 °C and a subsequent workup gave fascaplysin in an 80% yield. This method provides fascaplysin in a 72% overall yield from commercially available β-carboline.

**Scheme 9:**
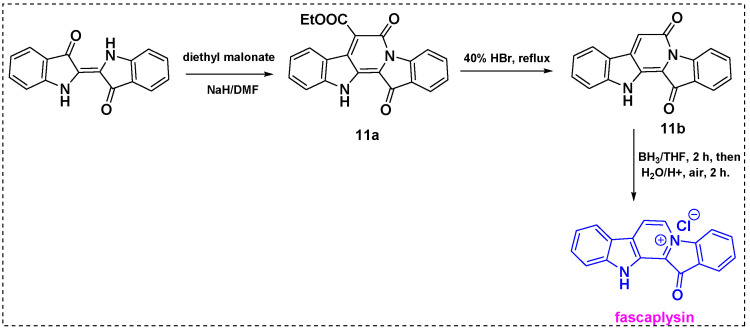
Total synthesis of fascaplysin from indigo.

The same research group reported a novel three-step method involving indigo and methylene active compounds for the total synthesis of fascaplysin and its derivatives in 2018 (Scheme 9) [31]. Indigo was reacted with diethyl malonate in DMF/NaH to produce **11a** in a 75% yield. Subsequent hydrolysis and decarboxylation of **11a** via refluxing in 40% hydrobromic acid for 2 h gave **11b** in a 95% yield. Finally, **11b** was refluxed with a BH_3_/THF complex for 2 h and then oxidized in air to afford fascaplysin in a 43% yield and, overall, a 71% yield from indigo.

**Scheme 10:**
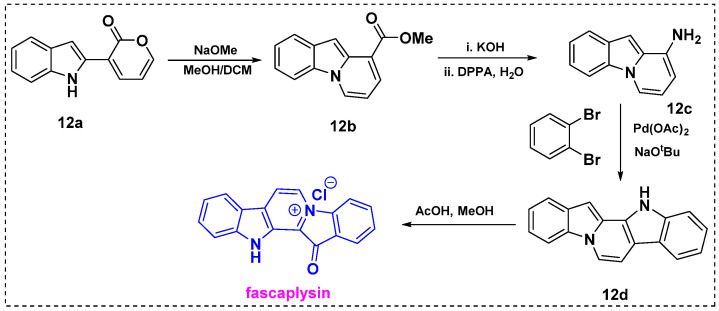
Formal synthesis of fascaplysin via pyrone remodeling.

In 2020, Sarpong et al. described the synthesis of diverse N-fused heterocycles, including the pyrido[1,2-*a*] indole scaffold, using an effective pyrone remodeling strategy [32]. To demonstrate its utility, this methodology was applied in the synthesis of fascaplysin (Scheme 10). Initially, indole–pyrone **12a** was reacted with sodium methoxide as a nucleophile in dichloromethane/methanol to furnish **12b** in a 61% yield. Then, hydrolysis of ester **12a** with KOH provided an intermediate carboxylic acid in a 99% yield, which efficiently underwent Curtius rearrangement in the presence of DPPA/H_2_O to generate amine **12c** in a 94% yield. Next, the palladium-catalyzed amination/C–H arylation domino coupling reaction of **12c** and 1,2-dibromobenzene in the presence of dppf ligand gave the product **12d** at 55%, which was further converted to fascaplysin via a previously reported procedure using acetic acid/methanol.

**Scheme 11:**
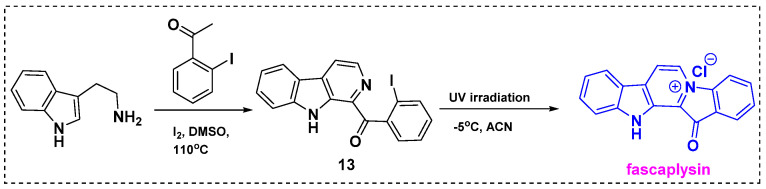
Total synthesis of fascaplysin from tryptamine by UV irradiation.

In 2023, Tryapkin et al. described a novel two-step synthesis of fascaplysin derivatives based on low-temperature UV quaternization (Scheme 11) [33]. The first step of this method was a modification of the synthetic routes from tryptamine and acetophenones previously reported by Zhu et al. and Battini et al. The reaction of tryptamine with 2-iodoacetophenone in DMSO/I_2_ at 110 °C gave isoquinoline **13** in a 40% yield. Next, UV quaternization was performed in acetonitrile at −5 °C to prevent side product formation, and fascaplysin was obtained in a 50% yield. The usage of acetonitrile with the temperature decreasing to −50 °C hinders the side reactions to allow obtaining fascaplysin in a good yield. Notably, only the starting material and quaternization product (fascaplysin) were observed in the reaction mixture. After fascaplysin was isolated, three-times-repeated irradiation of the starting material remaining in the reaction mixture increased the product yield to 91%.

### 2.2. Total and Formal Synthesis of Homofascaplysins B–C

The therapeutic potential of homofascaplysins A–C is based on a broad range of bioactivities, including anticancer, antibacterial, antifungal, antiviral, and antimalarial properties. Homofascaplysins A–C have effective cytotoxicity against L-1210 mouse leukemia cells and potent antimicrobial activity [7,34]. Although fascaplysin has been synthesized by several groups, as detailed in Section 1, only a few syntheses of homofascaplysins B and C have been reported. A total of five synthetic routes have been reported for total synthesis of fascaplysin C and three synthetic routes for homfascaplysin B since 1992. Here, we are going to briefly discuss each and every total synthesis of homofascaplysins A–C reported in the literature up to 2023.

**Scheme 12:**
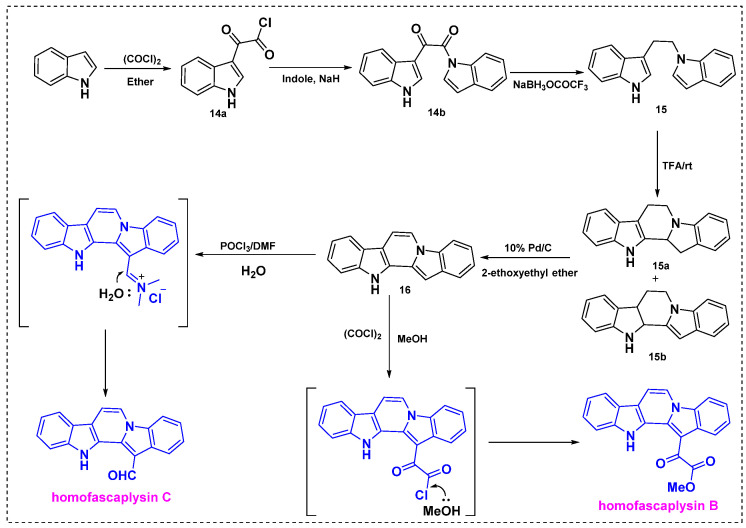
First total synthesis of homofascaplysins B and C from indole.

The first total synthesis was described in 1992 by Gribble et al., who obtained homofascaplysins B and C from indole in seven steps with overall yields of 76% and 67%, respectively (Scheme 12) [35]. These compounds were synthesized by peracid oxidation followed by reaction of the key diindole intermediate with oxalyl chloride/methanol or Vilsmeier formylation. In the first step, indole was reacted with oxalyl chloride under mild reaction conditions to give 3-indolyl glyoxylyl chloride **14a**, which was further treated with the sodium salt of indole to afford keto amide **14b** in an 86% yield. Treatment of keto amide **14b** with sodium trifluoroacetoxyborohydride provided diindole **15** in a 47% overall yield. Next, the neat reaction of diindole **15** in CF_3_CO_2_H at room temperature provided a 10:1 mixture of **15a** and **15b** in a good yield. The crude mixture of **15a** and **15b** was dehydrogenated with 10% Pd/C in EtOAc under reflux to give diindole **16** in a 98% yield. Finally, **16** was treated with the Vilsmeier reagent POCl_3_/DMF to obtain homofascaplysin C in an 88% yield or with oxalyl chloride followed by methanol to obtain homofascaplysin B in a 99% yield. In both reactions, intermediate **16** provided the regioselective products homofascaplysins B and C.

**Scheme 13:**
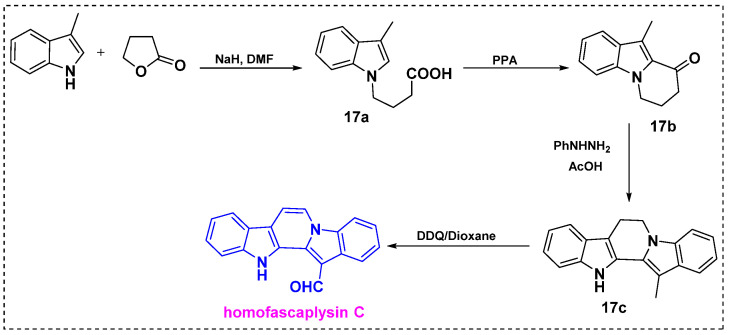
Total synthesis of homofascaplysin C from 3-methylindole.

In 1996, Dubovitskii et al. developed an efficient protocol for the total synthesis of homofascaplysin C from commercially available 3-methylindole in only four steps (Scheme 13) [36]. In the first step, the reaction of 3-methylindole with γ-butyrolactone in the presence of NaH/DMF provided acid **17a** in a 95% yield. Next, cyclization of acid **17a** in PPA at 100 °C gave pyrido[1,2-a] indole **17b** in a 90% yield. Using the Fischer indole synthesis method, **17b** was treated with phenylhydrazine hydrochloride in refluxing acetic acid to obtain 6,7-dihydro-13-methyl-12H-pyrido[1,2-a:3,4-b′]diindole **17c** in a 91% yield. Finally, oxidative dehydrogenation and oxidation of the methyl group to a formyl group by DDQ in dioxane under reflux for 2 h gave homofascaplysin C in a 50% yield and an 81% overall yield from the 3-methyl indole in four steps. This route is highly favorable for scaling up reactions because of the easy availability of the starting materials, like 3-methyl-indole.

**Scheme 14:**
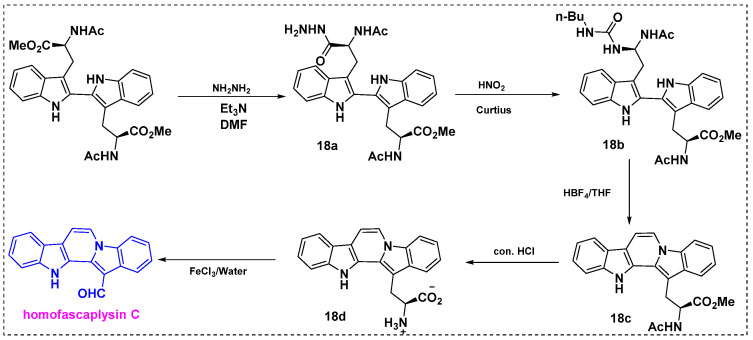
Total synthesis of homofascaplysin C from ditryptophan esters.

In 1999, Van Vranken et al. described the total synthesis of homofascaplysin C from peptides via the Mannich dimerization of tryptophan side chains (Scheme 14) [37]. This novel and efficient method relies on peptide chemistry. Initially, ditryptophan was converted into monohydrazide **18a** using 1.5 equiv of hydrazine in DMF, and the product yield was 30%. Triethylamine was added to the reaction mixture to facilitate proton-transfer steps. Subsequently, acyl hydrazide **18a** was converted into an acyl azide using a mixture of NaNO_2_, 1 N HCl, and glacial acetic acid in chloroform. After extraction and drying, the acyl azide was trapped by n-butylamine to give urea **18b** in a 48% yield. Unexpectedly, ionization of 18b in THF with anhydrous HBF_4_ gave the cyclized product **18c** in an 83% yield instead of the expected product of uncyclized aldehyde. The structure of **18c** was interesting because it contained the indolo[1,2-a] carbazole ring system found in fascaplysins. Finally, compound 18c was hydrolyzed to amino acid **18d**. The reaction was first performed under basic conditions, giving **18d** only in a 25% yield, but it was later found that the corresponding carboxamide hydrolyses under acidic conditions could give **18d** in a 43% yield, and then oxidative cleavage of the amino acid moiety with aqueous ferric chloride provided homofascaplysin C in a 23% yield, and the overall yield was 45% from tryptophan.

**Scheme 15:**
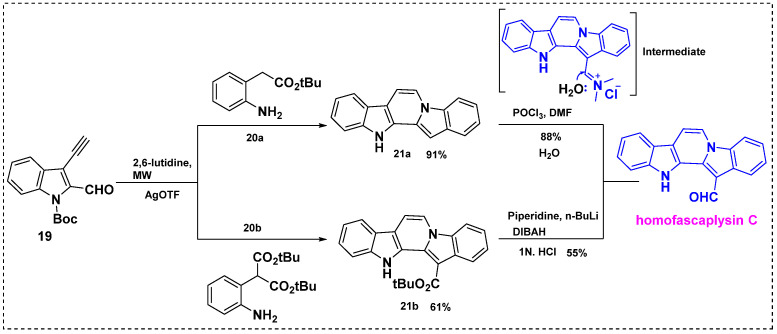
Total synthesis of homofascaplysin C via a silver-catalyzed cascade reaction.

In 2010, Waldmann et al. described a silver-catalyzed cascade reaction and its application in the total synthesis of homofascaplysin C from Boc-protected 3-ethynyl-indole-2-carbaldehyde **19** (Scheme 15) [27]. The microwave-assisted silver-catalyzed cascade cyclization of **19** with aniline **20a** or **20b** yielded pentacyclic core **21a** or **21b** in a 91% yield or a 61% yield, respectively. Partial reduction of the tert-butyl ester of **21b** with in situ generated lithium diisobutyl piperidino hydroaluminate provided homofascaplysin C in a 55% yield and a 58% overall yield from **19** (two steps). In contrast, diindole **21a** was effectively converted into homofascaplysin C in an 88% yield by formylation with POCl_3_ in DMF, and the overall yield was 89% from **19** (two steps). This reaction proceeded regioselectively to produce homofascaplysin C, and no other isomers were observed, as shown in Scheme 15.

**Scheme 16:**
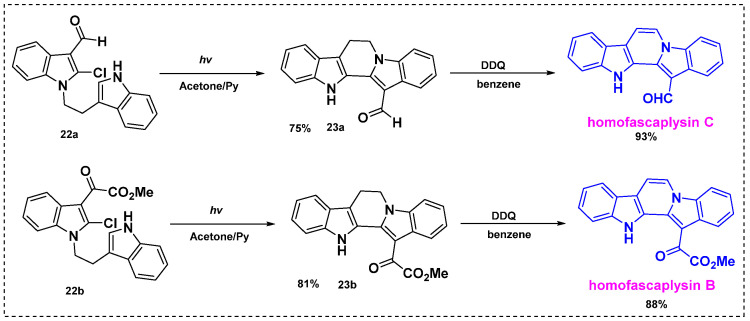
Total synthesis of homofascaplysins B and C through photocyclization/dehydrogenation reactions.

In 2012, Zhang et al. developed a two-step photocyclization/dehydrogenation reaction for the synthesis of homofascaplysins B and C from indole derivatives (Scheme 16) [38]. Irradiation of 2-chloro-1-[2-(indol-3-yl)-ethyl] indole-3-carbaldehyde 22a or 22b in deaerated anhydrous acetone containing pyridine at an ambient temperature for 12–13 h provided photocyclized products **23a** in a 75% yield or **23b** in an 81% yield. Subsequent dehydrogenation of **23a** or **23b** by DDQ in benzene for 2–3 h at room temperature gave homofascaplysins C and B in 93% and 88% yields, respectively. The overall yield was 84% for homofascaplysin C from **22a** and 84% for homofascaplysin C from **22b**. This group also developed a one-step sequential photocyclization and photochemical dehydrogenation reaction in Cu(OAc)_2_- and air-saturated acetone to synthesize homofascaplysins C and B directly from **22a** or **22b**, but the yields were very low. Therefore, the two-step photocyclization/dehydrogenation route is more efficient for the synthesis of homofascaplysins B and C and other analogs.

**Scheme 17:**
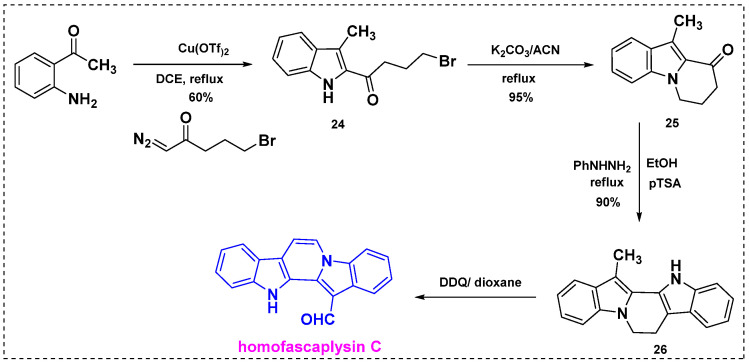
Total synthesis of homofascaplysin C from α-diazoketones.

In 2013, Reddy et al. applied an α-diazoketone-based methodology to the formal synthesis of homofascaplysin C [39]. Initially, 2,4,5-trisubstituted pyrrole derivatives were synthesized by coupling α-diazoketones with β-enaminoketones and esters using 10 mol% Cu(OTf)_2_. In addition, a wide range of 2,3-disubstituted indole derivatives were prepared from α-diazoketones and 2-aminoaryl or alkyl ketones. Finally, homofascaplysin C was synthesized by coupling an alkyl diazoketone with 2-aminoacetophenone (Scheme 17). Specifically, the coupling of 2-aminoacetophenone with bromo butyric diazoketone in the presence of 10% Cu(OTf)_2_ afforded 2,3-disubstituted indole **24** in a 60% yield. Subsequent intramolecular cyclization of **24** in K_2_CO_3_/acetonitrile under reflux afforded compound **25** in a 95% yield. Further treatment of **25** with phenylhydrazine in the presence of pTSA in ethanol at 80 °C gave Fischer indole product **26** regioselectively in a 90% yield. The oxidation of **26** under the conventional DDQ/dioxane conditions reported in the literature provided homofascaplysin C in a 50% yield and a 74% overall yield from 2-amino acetophenone in four steps.

**Scheme 18:**
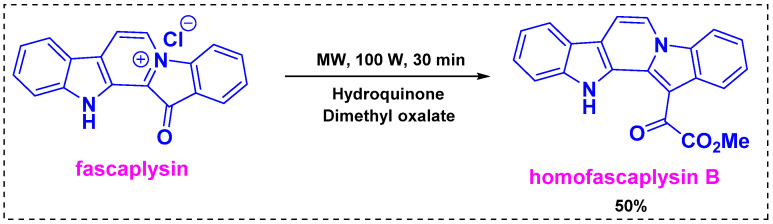
One-step transformation of the marine alkaloid fascaplysin into homofascaplysin B.

In 2018, Zhidkov et al. developed a one-step transformation of the marine alkaloid fascaplysin into homofascaplysin B using traditional heating or microwave conditions [40]. In the heating method, the reaction of fascaplysin with excess dimethyl oxalate and Na_2_S_2_O_3_ (10 equiv) in an autoclave at 200 °C for 30 min produced homofascaplysin B in a 30% yield. The presence of Na_2_S_2_O_3_ (10 equiv) increased the yield from 24% to 30%. To further improve the yield, microwave irradiation was employed. Under irradiation at 100 W, the reaction of fascaplysin with dimethyl oxalate was complete after 30 min and provided a similar yield of homofascaplysin B. Subsequently, fascaplysin was reacted with excess dimethyl oxalate under microwave irradiation at 100 W for 30 min in the presence of dihydroquinone as a reducing agent, and these optimal conditions provided homofascaplysin B in a 50% yield (Scheme 18). The mechanism of this reaction was not investigated properly in the manuscript, but the reaction proceeded regioselectively.

## 3. Conclusions

Owing to its anticancer and antimicrobial properties, fascaplysin and its derivatives have potential for therapeutic applications. Moreover, fascaplysin exhibits potent antiplasmodial, antioxidant, and anti-inflammatory activities, highlighting new paths for the development of biomedical applications and paving the way for developing novel fascaplysin analogs. All the above discussed methods except Bharate et al.’s method have some drawbacks like harsh reaction conditions, difficulty scaling up, and limited fascaplysin analog synthesis. The total synthesis developed by Bharate et al. has significant scope for scaling up to the kilogram scale and synthesis of various fascaplysin derivatives using commercially available starting materials.

However, owing to its unusual planar structure, fascaplysin can intercalate in DNA, which results in extreme toxicity and may affect drug product formation. To reduce these toxic effects, the development of related targeted drugs via the structural modification of fascaplysin is essential. Surprisingly, no synthetic methods have yet been reported for homofascaplysin A. In our opinion, development of a new synthetic route to total synthesis of homofascaplysin A and its derivatives is worth further study in terms of its DNA intercalation and toxic effects. This review, which summarizes the advances in the total synthesis of fascaplysin and its metabolites over the past 30 years, will aid in promoting the development of efficient, scalable, and ecofriendly methods for generating these compounds as well as new derivatives.

## Data Availability

References are given to support the data presented in this study.
